# Current high prevalences of *Strongyloides stercoralis* and *Opisthorchis viverrini* infections in rural communities in northeast Thailand and associated risk factors

**DOI:** 10.1186/s12889-018-5871-1

**Published:** 2018-07-31

**Authors:** Pokkamol Laoraksawong, Oranuch Sanpool, Rutchanee Rodpai, Tongjit Thanchomnang, Wanida Kanarkard, Wanchai Maleewong, Ratthaphol Kraiklang, Pewpan M. Intapan

**Affiliations:** 1 Department of Public Health Administration, Health Promotion, Nutrition, Faculty of Public Health, Khon Kaen University, Khon Kaen, Thailand; 2 Department of Parasitology, Faculty of Medicine, Khon Kaen University, Khon Kaen, Thailand; 3 Faculty of Medicine, Mahasarakham University, Mahasarakham, Thailand; 4 Department of Computer Engineering, Faculty of Engineering, Khon Kaen University, Khon Kaen, Thailand; 5 Research and Diagnostic Center for Emerging Infectious Diseases, Khon Kaen University, Khon Kaen, Thailand

**Keywords:** Prevalence, Intestinal parasites, *Strongyloides stercoralis*, *Opisthorchis viverrini*, Thailand

## Abstract

**Background:**

Two important helminths, *Strongyloides stercoralis* (an intestinal roundworm) and *Opisthorchis viverrini* (a liver fluke), are endemic in northeast Thailand. There have been national campaigns in place aimed at the control and eradication of soil-transmitted helminthiasis and opisthorchiasis in Thailand for several decades. However, these helminths still exist and raise concerns regarding public health. This study aimed to evaluate the current prevalence of *S. stercoralis* and *O. viverrini* infections in rural communities in northeast Thailand. The data from this study will be useful to improve strategies for future helminth prevention and control.

**Methods:**

A cross-sectional study was conducted from December 2016 to June 2017 in Mueang Khon Kaen district in Khon Kaen, Thailand. The participants were selected using a simple random sampling method. Demographic data were collected using a questionnaire. Stool samples were collected and processed using agar plate culture to determine the presence of *S. stercoralis* infection and an in-house formalin-ethyl acetate concentration technique to determine the presence of *O. viverrini* and other intestinal parasite infections (IPIs).

**Results:**

In total, 602 persons were enrolled. However, only 526 were analyzed for *S. stercoralis* and 387 for *O. viverrini* risk factors. The overall prevalence of *S. stercoralis* infection was 23.0% (95% confidence interval [95%CI]: 19.4 to 26.6). The prevalence of *O. viverrini* infection and IPIs other than *S. stercoralis* was 20.4% (95%CI: 16.5 to 24.8). The prevalence of *O. viverrini* infection was 19.4% (95%CI: 15.6 to 23.7). Male sex was significantly associated with *S. stercoralis* infection [Adjusted Odds Ratio (aOR) 4.0; 95%CI: 2.5 to 6.2; *P-value* < 0.001]. Males were significantly more likely to be infected with *O. viverrini* and other IPIs (aOR 4.1; 95%CI: 2.3 to 7.2, *P-value* < 0.001).

**Conclusions:**

This study demonstrated that the updated prevalence of intestinal parasite infections is still high in rural communities in northeast Thailand, especially that of strongyloidiasis and opisthorchiasis.

## Background

Human strongyloidiasis is a soil-transmitted helminthiasis caused by *Strongyloides stercoralis.* It is widespread throughout Southeast Asia, including in Thailand [1–5]. It occurs primarily in developing communities and is often found in travelers and former war veterans, immigrants, immunocompromised inhabitants, and people exposed to soil [6]. Some patients presented gastro-intestinal symptoms, dermatological symptoms, hyperinfection, or disseminated strongyloidiasis, which can involve several organs and lead to fatal outcomes [7, 8]. Additionally, chronic strongyloidiasis can lead to malnutrition in children and adolescents, causing growth retardation [7]. The human liver fluke (*Opisthorchis viverrini*) is a cause of critical public health problems in some areas of Southeast Asia including Lao People’s Democratic Republic (Lao PDR), Cambodia, central Vietnam, and Thailand [9]. *Opisthorchis viverrini* is classified as a Group 1 carcinogen by the World Health Organization (WHO) [10] and is associated with hepatobiliary diseases and the etiological agents of bile duct cancer (cholangiocarcinoma). For several decades there have been intensive national control programs aimed at combating soil-transmitted helminths and opisthorchiasis in rural Thailand [11, 12]. Moreover, there have been great initiatives put into place to control the *O. viverrini* infection and the resulting cholangiocarcinoma including the EcoHealth/One Health approach (the Lawa Lake control approach) [13] and the Cholangiocarcinoma Screening and Care Program [14]. This study examines the current prevalences of *S. stercoralis* and *O. viverrini* infections in rural communities in the Mueang Khon Kaen district in Khon Kaen, Thailand. Intestinal parasitic infections other than *S. stercoralis* and *O. viverrini* were also reported. This result is important for monitoring and the implementation of effective control strategies.

## Methods

### Study design

**Fig. 1:**
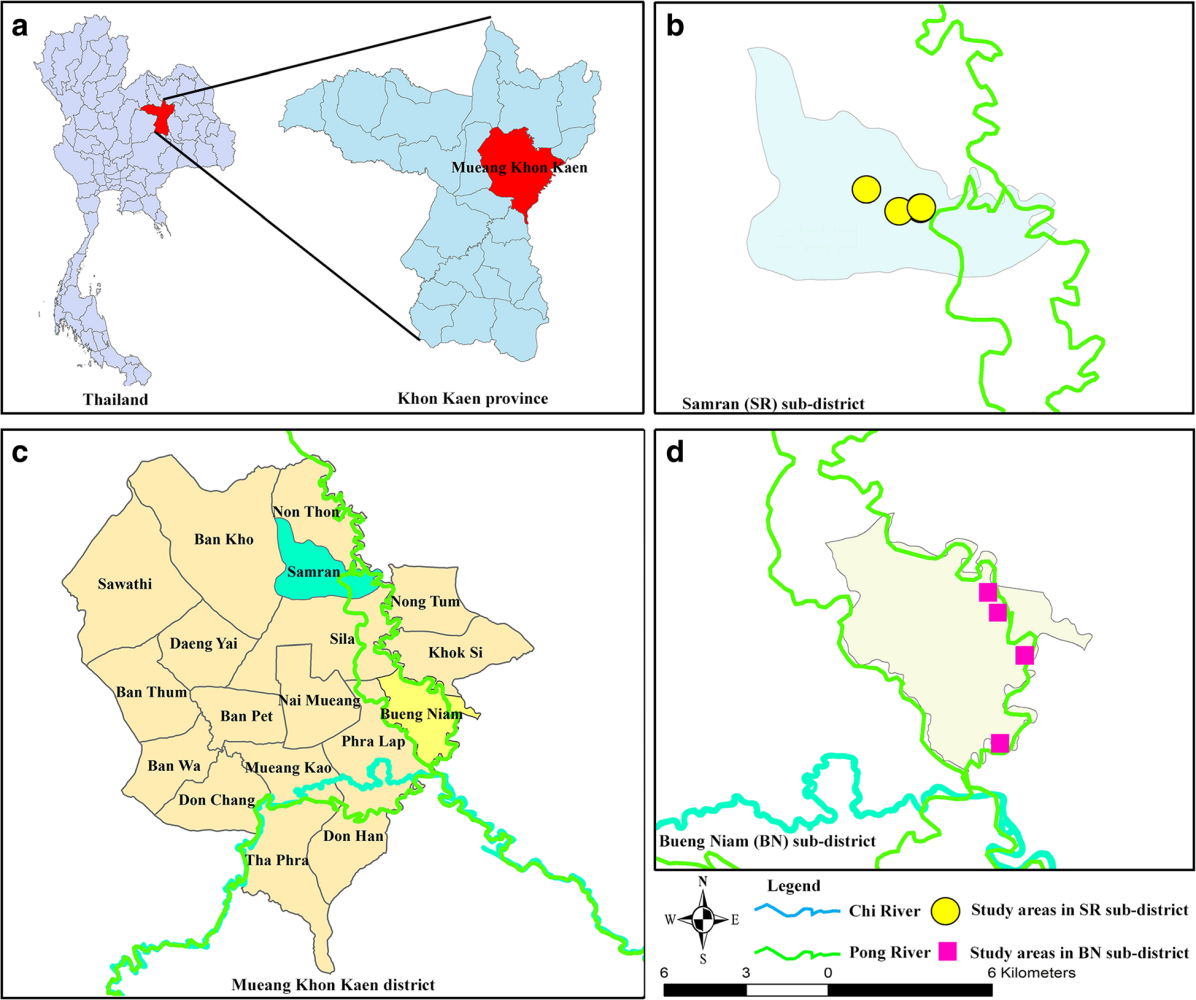
(**a**) indicates Khon Kaen province, located in northeast Thailand, and indicates the Mueang Khon Kaen District located in Khon Kaen Province. (**b**) indicates the Samran (SR) and Bueng Niam (BN) sub-districts, located in Mueang Khon Kaen District. (**c** and **d**) indicate areas in the SR and BN sub-districts in which samples were collected, respectively

This cross-sectional survey was carried out from December 2016 to June 2017 in two sub-districts in Mueang Khon Kaen district in Khon Kaen, Thailand. Mueang Khon Kaen is located at 8.71944^๐^ latitude and 99.791667^๐^ longitude (Fig. 1). The average temperature was 27.7 °C (16.0 °C-39.9 °C) with an annual rainfall of 1031.4 mm [15]. Factors associated with the tropical environment in this area (especially education, socio-cultural factors, economic factors, climate, land use, rivers, and rainfall) affect the prevalence of intestinal parasite infection (including that of *S. stercoralis* and *O. viverrini*) [16, 17]. The Mueang Khon Kaen District was selected as the study site because it has a higher prevalence of strongyloidiasis than any other district in Khon Kaen [17]. People in this area commonly eat raw, pickled, or undercooked fish and other aquatic animals, which are foods that carry a high risk of *O. viverrini* infection [18]. We randomly selected villages along the Pong River classified by their varied flooding patterns. Most of the land in two sub-districts is used for farming. Agriculture is the primary economic activity of the population in these areas [15].

### Study population, sample size, and sampling technique

**Fig. 2:**
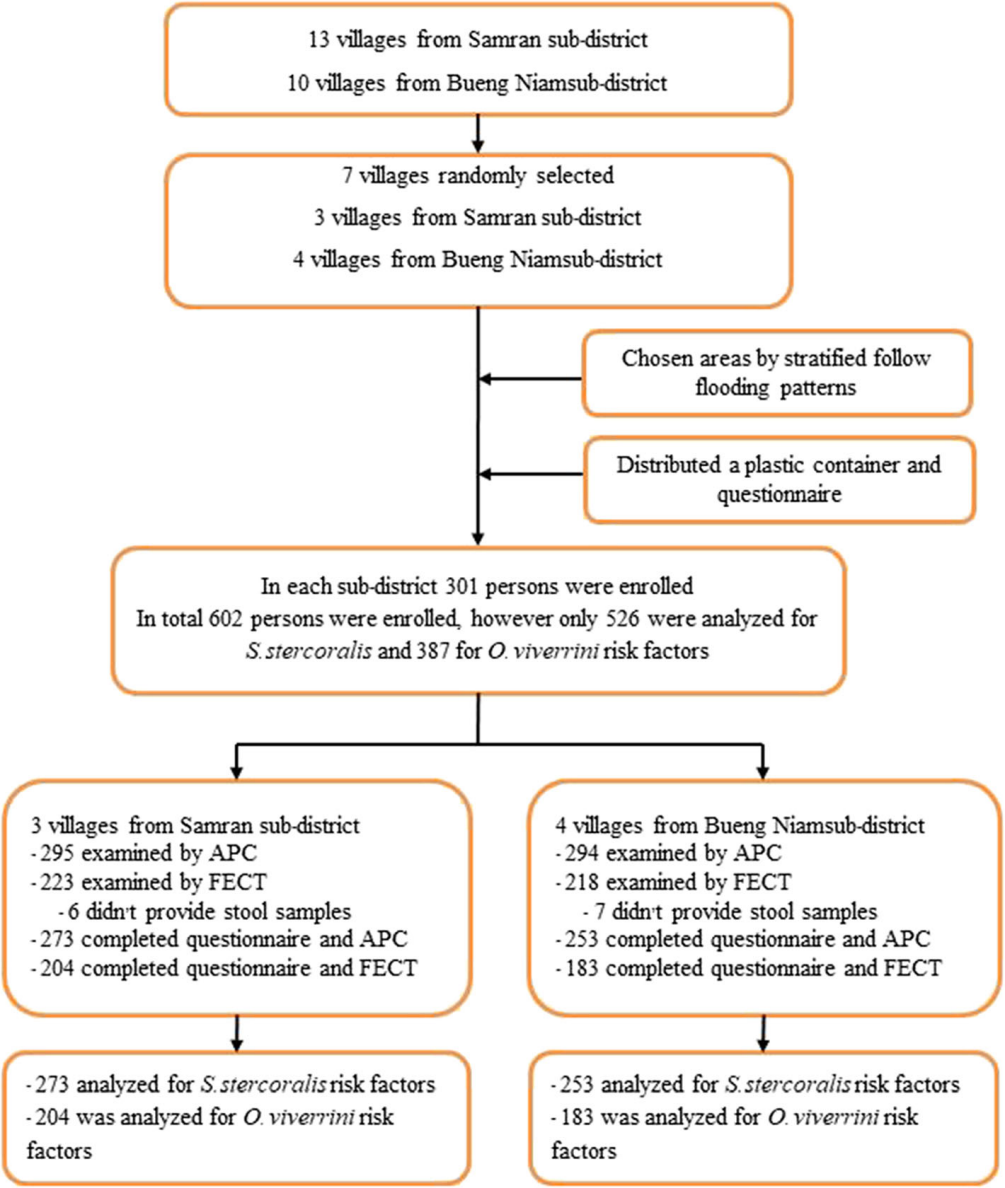
Flow chart of the study’s activities

**Fig. 3:**
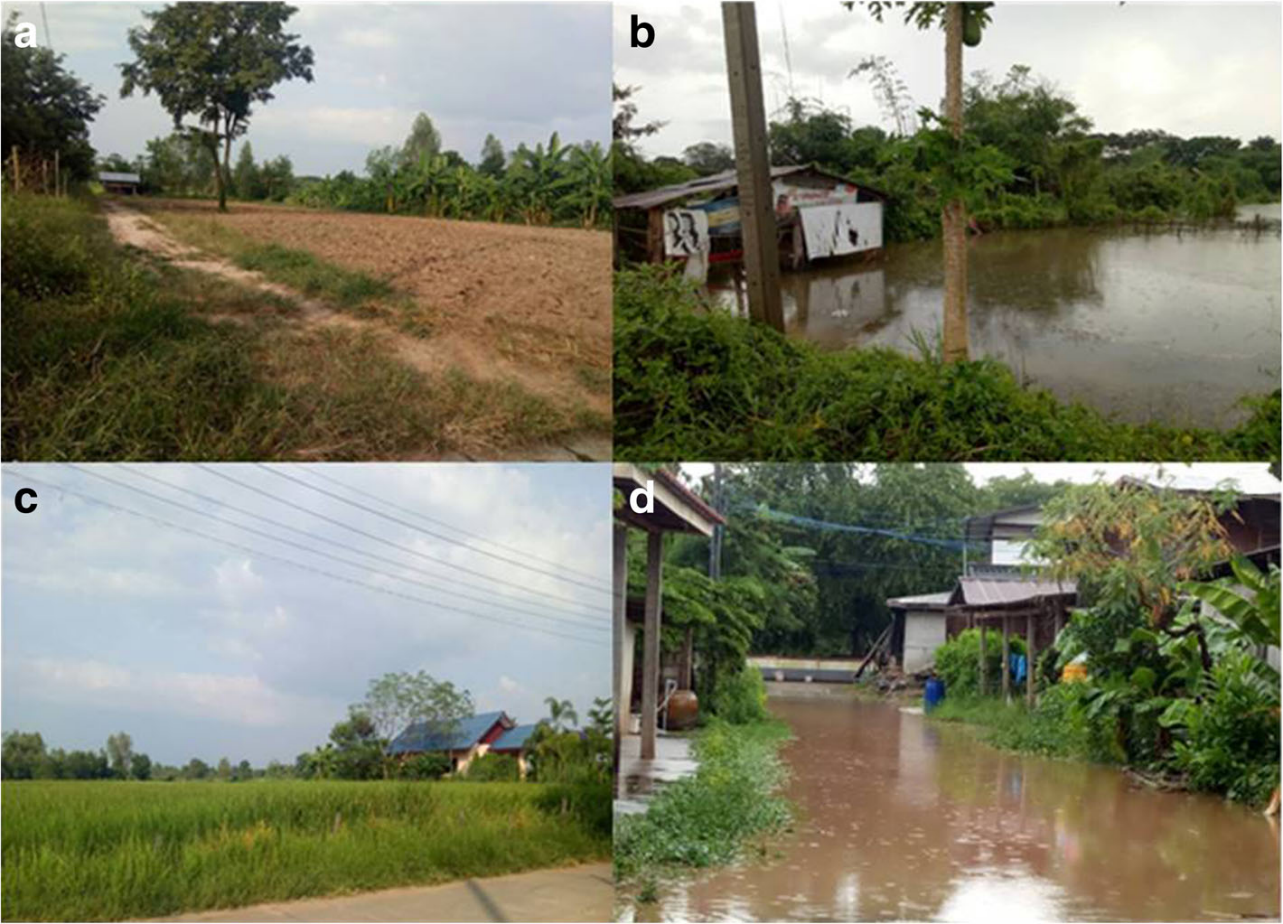
Geographic characteristics of Samran (SR) and Bueng Niam (BN) sub-districts; **a** and **c** show the geographic characteristics of the SR sub-district during the rainy season, **b** and **d** show the geographic characteristics of the BN sub-district during the rainy season

We selected two sub-districts in the Mueang Khon Kaen district to conduct this study: Samran (SR), which had no history of flooding, and Bueng Niam (BN), which experiences at least one flood per year. These areas were selected based on data from the hospital-based study. It was found that the Mueng Khon Kaen district has the highest prevalence of strongyloidiasis than other districts in Khon Kaen [17]. The sample size was determined using the single population proportion formula [19]. It was calculated using a prevalence rate (p) of 23.0%, as detailed in a previous study [1], with a 95% confidence interval (z = 1.96) and a 5% margin of error (d = 0.05). The calculated sample size was 273 people per sub-district. We assumed that the final sample size would end up being reduced by around 10% due to subjects being unable to pass stool on the study date. Thus, we aimed for a sample size of 301 per sub-district. A simple random sampling method was used to select the population from each sub-district. Inclusion criteria were participants who were ≥ 8 years old and lived in both areas. The individual was randomly selected by the simple random sampling method. We randomly selected 301 people per area and then gave them instructions and distributed plastic containers for stool collection. A total of 589 subjects (295/50.08%) of the total population of three villages in SR and 49.92% (294) from that of four villages in BN returned stool specimens (Fig. 2; Fig. 3).

### Data collection and laboratory processing

Field staff visited randomly selected areas of the two sub-districts accompanied by the local public health and primary health care officers center. After receiving informed consent from the participants, authorization from parents or legal guardians of minor participants was obtained. The questionnaire included baseline characteristics, knowledge about *S. stercoralis* and *O. viverrini,* and risk behaviors for both parasite infections (contact soil, consume raw fish). A total of 589 participants were interviewed by a self-administered questionnaire, or by parents or legal guardians of minors. Then, stool samples were collected. Before stool-sample collection, a cleaned plastic container labeled with the participant’s name and identification was distributed to each participant by the village health volunteers. One day later, they returned the full container to the field staff. The containers were transported immediately to the parasitology laboratory at the Khon Kaen University Faculty of Medicine. Before data were analyzed, the data were entered by two researchers for double-checking the quality of the data.

Agar plate culture (APC) [20] was used to detect *S. stercoralis* in each specimen, and the in-house formalin-ethyl acetate concentration technique (FECT) [21] was used to examine *O. viverrini* and other intestinal parasites. Two to four grams of stool from each sample was placed on 1.5%nutrient agar plates and incubated at 25-27^๐^C for 3–5 days [20]. The agar plate examination was done under a stereo microscope on the third or fifth day. The formalin-ethyl acetate concentration technique was conducted as follows: two grams of stool from each of the samples was suspended in a container of 10 ml of normal saline solution. The suspension was strained through two layers of gauze into a 15-ml centrifuge tube and centrifuged at 700 x *g* for five minutes. The supernatant was then decanted. After that, 7 ml of 10% formalin was added to the sediment, mixed well, and allowed to stand for two minutes. Then, 3 ml of ethyl acetate was added. The tube was closed, vigorously shaken for one minute, and centrifuged at 700 x *g* for five minutes. The debris plug in the ethyl acetate layer was loosened, and the top three layers were discarded, leaving the sediment. One milliliter of 10% formalin was then added to re-suspend the sediment, which was examined for parasites under a compound microscope [21].

### Data analysis

Demographic characteristics of the participants are described using the frequency, percentage, mean, and standard deviation (SD). The prevalence of *S. stercoralis*, *O. viverrini*, and other intestinal parasitic infections (IPIs) is described as a proportion and 95%CI. To investigate factors that affect *S. stercoralis*, *O. viverrini*, and other IPIs, the prevalence rate and their 95%CIs were estimated using logistic regression and the generalized estimating equation for survey sampling. To adjust for possible confounders, all variables with a *P-value* less than 0.1 in the univariate analysis were selected. A *P-value* of less than 0.05 was considered statistically significant. The statistical analysis was conducted using the STATA package version 10.1. College Station, Texas: StataCorp LLC.

## Results

### Demographic characteristics

In total, 602 persons were enrolled. However, only 526 were analyzed for *S. stercoralis* and 387 for *O. viverrini* risk factors. A total of 526 participants were enrolled in this study (273 [51.9%] from the SR sub-district, 253 [48.1%] from the BN sub-district). Two-hundred fifty-four (48.3%) of the 526 participants were male, and 272 (51.7%) were female. The mean age (±SD) of participants was 55.9 (± 14.4; range = 11–91 years), and most of the participants were ≥ 60 years old (246/526; 46.8%). The most common occupation of participants was agriculture 368/526 (69.9%), and the majority had a primary-school education or no formal education 381/526 (72.4%). The average income (±SD) was 160.4 ± 168.1$ (0 to 1500.2$), and 397/526 participants (75.5%) had an income lower than $250 per month (according to the USD-THB exchange rate as of 1 November 2017), which is the poverty line in Thailand [22] (Table 1).

**Table 1:** Demographic characteristics of study participants in Mueang Khon Kaen district, Khon Kaen province, Thailand

Demographic data	SR		BN		Total	
	Number	Percentage	Number	Percentage	Number	Percentage
Gender (N)	273		253		526	
Female	144	52.8	128	50.6	272	51.7
Male	129	47.2	125	49.4	254	48.3
Age (N)	273		253		526	
< 30	18	6.6	10	4.0	28	5.3
30–39	17	6.2	12	4.7	29	5.5
40–49	45	16.5	51	20.2	96	18.3
50–59	68	24.9	59	23.3	127	24.1
≥ 60	125	45.8	121	47.8	246	46.8
Mean ± SD (range)	55.6 ± 14.8 (11 to 91)		56.2 ± 14.0 (12 to 80)		55.9 ± 14.4 (11 to 91)	
Education level (N)	273		253		526	
Diploma, bachelor degree or higher	17	6.2	12	4.7	29	5.5
Grade 7–12	57	20.9	59	23.3	116	22.1
Primary school or no formal education	199	72.9	182	72.0	381	72.4
Occupation (N)	273		253		526	
Student, government/private office	24	8.8	8	3.2	32	6.1
Employee/Merchant/older	93	34.1	33	13.0	126	24.0
Agriculturist	156	57.1	212	83.8	368	69.9
BMI (N)	259		245		504	
< 18.5	22	8.5	9	3.7	31	6.2
18.5 to 25.9	140	54.1	136	55.5	276	54.7
26 to 29.9	78	30.1	79	32.2	157	31.2
≥ 30	19	7.3	21	8.6	40	7.9
Mean ± SD (range)	24.0 ± 4.2 (12.9 to 38.1)		24.5 ± 4.1 (16.8 to 50.2)		24.2 ± 4.2 (12.9 to 50.2)	
Income per month (N) $	273		253		526	
< 250 $	192	70.3	205	81.0	397	75.5
≥ 250 $	81	29.7	48	19.0	129	24.5
Mean ± SD (range)	165.1 ± 179.8 (0 to 1200.1)		155.2 ± 154.6 (0 to 1515.2)		160.4 ± 168.1 (0 to 1500.2)	

*SR* Samran sub-district, *BN* Bueng Niam sub-district

### Prevalence of *S. stercoralis* infection as detected using the APC technique

The overall prevalence of *S. stercoralis* infection as detected using APC was 121/526 participants (23.0%; 95%CI: 19.4 to 26.6), 88 (16.7%) of whom were male and 33 (6.3%) of whom were female. Most of the participants infected with *S. stercoralis* (95/526; 18.0%) had a primary-school level or no formal education. Moreover, those working in agriculture had a higher prevalence of *S. stercoralis* infection than those in other occupations (95/526; 18.0%). Most participants infected with *S. stercoralis* had an income of less than $250.00 per month (97/526; 18.4%) (Table 2). In the SR sub-district, the prevalence of *S. stercoralis* infection was 61/273 (22.3%; 95% CI: 17.5 to 27.8). Forty-one (15.0%) of the people infected in this sub-district were male, and 20 (7.3%) were female. In the BN sub-district, the prevalence of *S. stercoralis* infection was 60/253 (23.7%; 95%CI: 18.6 to 29.4) participants, 47 (18.6%) of whom were male and 13 (5.1%) of whom were female. The prevalence of *S. stercoralis* infection increased with age in the overall and both sub-district samples (Table 2). The difference in prevalence of *S. stercoralis* infection between the two sub-districts was not statistically significant.

**Table 2:** Univariable and multivariable analysis of *S. stercoralis* infection on simple logistic regression and GEE

Characteristics	SR (*n* = 273)^a^		BN (*n* = 253)^a^		Total (*n* = 526)		Crude OR (95%CI)	Adjusted OR (95%CI)
	Total No.	Positive No. (%)	Total No.	Positive No. (%)	Total No.	Positive No. (%)		
Gender (N)	273	61 (22.3)	253	60 (23.7)	526	121 (23.0)		
Female	144	20 (7.3)	128	13 (5.1)	272	33 (6.3)	1	1
Male	129	41 (15.0)	125	47 (18.6)	254	88 (16.7)	3.8 (2.45 to 5.99)^#^	4.0 (2.52 to 6.24)^#^
Age (N)	273	61 (22.3)	253	60 (23.7)	526	121 (23.0)		
< 30	18	0 (0)	10	1 (0.4)	28	1 (0.2)	1	1
30–39	17	1 (0.4)	12	1 (0.4)	29	2 (0.4)	2.0 (0.17 to 23.38)	1.9 (0.16 to 22.64)
30–49	45	12 (4.4)	51	8 (3.2)	96	20 (3.8)	7.1 (0.91 to 55.51)	7.7 (0.97 to 61.43)
50–59	68	20 (7.3)	59	14 (5.5)	127	34 (6.5)	9.9 (1.29 to 75.47) ^+^	10.8 (1.38 to 84.11) ^+^
≥ 60	125	28 (10.3)	121	36 (14.2)	246	64 (12.2)	9.5 (1.26 to 71.29) ^+^	10.0 (1.31 to 76.43) ^+^
Education level (N)	273	61 (22.3)	253	60 (23.7)	526	121 (23.0)		
Diploma, bachelor degree or higher	17	0 (0)	12	2 (0.8)	29	2 (0.4)	1	
Grade 7–12	57	11 (4.0)	59	13 (5.1)	116	24 (4.6)	3.5 (0.78 to 15.86)	
Primary school or no formal education	199	50 (18.3)	182	45 (17.8)	381	95 (18.0)	4.5 (1.04 to 19.21)^+^	
Occupation (N)	273	61 (22.3)	253	60 (23.7)	526	121 (23.0)		
Low risk of contact with *S. stercolaris*	24	2 (0.7)	8	0 (0)	32	2 (0.4)	1	
Moderate risk of contact with *S. stercolaris*	93	21 (7.7)	33	3 (1.2)	126	24 (4.6)	3.5 (0.78 to 15.79)	
Higher risk of contact with *S. stercolaris*	156	38 (13.9)	212	57 (22.5)	368	95 (18.0)	5.2 (1.22 to 22.25) ^+^	
BMI (N)	259	56 (21.6)	245	59 (24.1)	504	115 (22.8)		
< 18.5	22	5 (1.9)	9	3 (1.2)	31	8 (1.6)	1	
18.5 to 25.99	140	34 (13.1)	136	46 (18.8)	276	73 (14.5)	1.0 (0.44 to 2.41)	
26 to 29.99	78	13 (5.0)	79	5 (2.0)	157	25 (5.0)	0.5 (0.21 to 1.35)	
≥ 30	19	4 (1.5)	21	5 (2.0)	40	9 (1.8)	0.8 (0.27 to 2.49)	
Income per month (N) $	273	61 (22.3)	253	60 (23.7)	526	121 (23.0)		
≥ 250 $	81	17 (6.2)	48	7 (2.8)	129	24 (4.6)	1	
< 250 $	192	44 (16.1)	205	53 (20.9)	397	97 (18.4)	1.4 (0.85 to 2.33)	

Remark: Those with a low risk of contact with *S. stercoralis* included students and government and private officers. Those with moderate risk of contact with *S. stercoralis* were employee/merchants and housewife. Those with a higher risk of contact with *S. stercoralis* were agriculturists. GEE: generalized estimating equation, SR: Samran sub-district; BN: Bueng Niam sub-district. Gender, age, education level, and occupation were multivariable analysis to adjust for confounder

^#^Statistically significant difference *P*-value < 0.001, ^+^Statistically significant difference P-value < 0.05

aThe difference of *S. stercoralis* infection between SR and BN was not statistically significant

### Risk factors associated with *S. stercoralis* infection

According to univariate analysis, demographic characteristics associated with *S. stercoralis* infection were sex, age, education level, and occupation. Males were 3.8 times more likely to be infected with *S. stercoralis* than females (crude Odds Ratio [cOR] 3.8; 95%CI: 2.45 to 5.99, *P*-value < 0.001). Patients 50–59 years of age and ≥ 60 years were 9.9 and 9.5 times more likely to be infected with *S. stercoralis* than those of other age groups, respectively (cOR 9.9; 95%CI: 1.29 to 75.47, cOR 9.5; 95%CI: 1.26 to 71.29, *P-*value < 0.003, respectively). Participants with a primary school level or no formal education were 4.5 times more likely than those with a diploma, bachelor’s degree, or higher to be infected with *S. stercoralis* (cOR 4.5; 95%CI: 1.04 to 19.21, *P-*value = 0.037). Additionally, agriculturists were 5.2 time more likely to be infected with *S. stercoralis* than participants in other occupations (cOR 5.2; 95%CI: 1.22 to 22.25, *P-*value = 0.009). Body mass index and income were not significantly associated with *S. stercoralis* infection (*P-*value > 0.05). After multivariable analysis to adjust for possible confounders, gender and age were found to be risk factors associated with *S. stercoralis* infection. Males were 4.0 times more likely to be infected with *S. stercoralis* than females (aOR 4.0; 95%CI: 2.52 to 6.24, *P-*value < 0.001; Table 2).

### Prevalence of *O. viverrini* and IPIs other than *S. stercoralis* detected using FECT

**Fig. 4:**
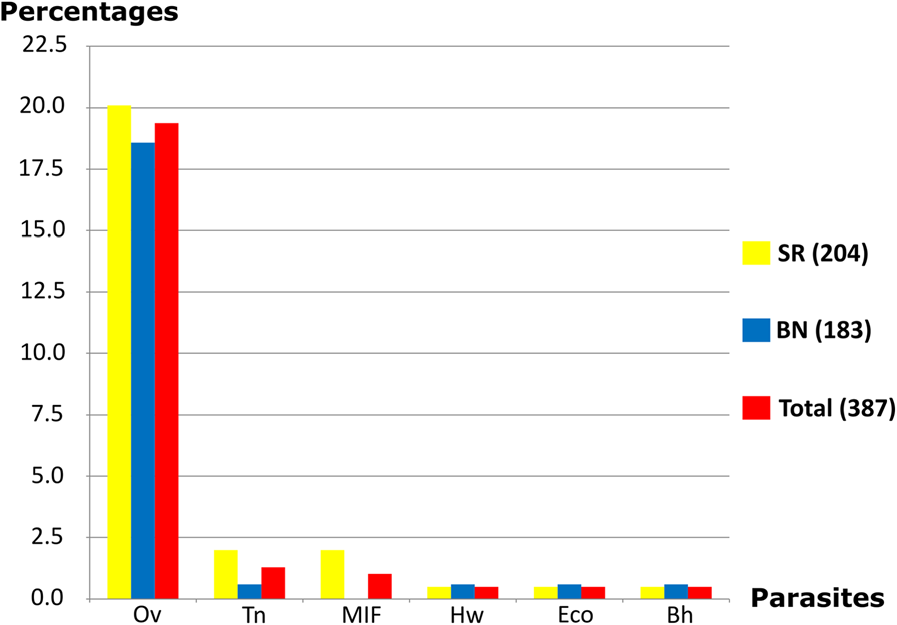
Prevalence of intestinal parasitic infections except *Strongyloides stercoralis* examined using FECT in Mueang Khon Kaen district, Khon Kaen province, Thailand, classified by parasite. Remark: Bh; *Blastocystis hominis*, Eco; *Entamoeba coli*, Hw; Hookworms, MIF; Minute Intestinal Flukes, Ov; *Opisthorchis viverrini*, Tn; *Taenia* spp. FECT: formalin ethyl acetate concentration technique

The 387 stool samples that remained after APC examination were subjected to FECT. The overall prevalence of *O. viverrini* and IPIs other than *S. stercoralis* was 79/387 (20.4%; 95%CI: 16.5 to 24.8). The highest prevalence of infection was of *O. viverrini* infection, which was 75/387 (19.4%; 95%CI: 15.6 to 23.7), followed by *Taenia* spp., which was 5/387 (1.3%; 95%CI: 0.4 to 3.0; Fig. 4). In the SR sub-district, the prevalence of *O. viverrini* and other IPIs was 45/204 (22.1%). In the BN sub-district, the prevalence of *O. viverrini* and other IPIs was 34/183 (18.6%). The prevalence of *O. viverrini* and other IPIs did not differ significantly between the two sub-districts (Table 3).

**Table 3:** Univariable and multivariable analysis of *O. viverrini* and other IPs infections on simple logistic regression and GEE

Characteristics	SR^a^		BN^a^		Total		Crude OR	Adjusted OR
	Total No.	Positive No. (%)	Total No.	Positive No. (%)	(95%CI)	Positive No. (%)	(95%CI)	(95%CI)
Gender (N)	204	45 (22.1)	183	34 (18.6)	387	79 (20.4)		
Female	114	13 (6.4)	98	17 (9.3)	212	30 (7.7)	1	1
Male	90	32 (15.7)	85	17 (9.3)	175	49 (12.7)	2.4 (1.4 to 3.9)^#^	4.1 (2.3 to 7.2)^#^
Age (N)	204	45 (22.1)	183	34 (18.6)	387	79 (20.4)		
< 30	12	2 (1.0)	5	0 (0)	17	2 (0.5)	1	1
30–39	8	2 (1.0)	6	1 (0.5)	14	3 (0.8)	2.0 (0.3 to 14.4)	1 (N/A)
30–49	34	6 (2.9)	39	6 (3.3)	73	12 (3.1)	1.5 (0.3 to 7.3)	5.8 (0.6 to 60.0)
50–59	51	9 (4.4)	43	8 (4.4)	94	17 (4.4)	1.6 (0.3 to 7.9)	7.6 (0.7 to 77.3)
≥ 60	99	26 (12.8)	90	19 (10.4)	189	45 (11.6)	2.3 (0.5 to 10.6)	4.5 (0.4 to 45.0)
Education level (N)	204	45 (22.1)	183	34 (18.6)	387	79 (20.4)		
Diploma, bachelor degree or higher	10	1 (0.5)	7	1 (0.5)	17	2 (0.5)	1	1 (empty)
Grade 7–12 or diploma	46	7 (3.4)	39	2 (1.1)	85	9 (2.3)	0.9 (0.2 to 4.5)^**+**^	0.8 (0.4 to 1.7)
Primary school or no formal education	148	37 (18.1)	137	31 (16.9)	285	68 (17.6)	2.3 (0.5 to 10.5)^**+**^	1 (omitted)
Occupation (N)	204	45 (22.1)	183	34 (18.6)	387	79 (20.4)		
Student, government/private officer	17	4 (2.0)	4	0 (0)	21	4 (1.0)	1	1
Employee/Merchant	41	6 (3.0)	19	3 (1.6)	60	9 (2.3)	0.8 (0.2 to 2.8)	1.6 (0.2 to 16.2)
Housewife	24	9 (4.4)	2	0 (0)	26	9 (2.3)	2.2 (0.6 to 8.7)	13.3 (1.3 to 137.5)^+^
Agriculturist	122	26 (12.7)	158	31 (16.9)	280	57 (14.7)	1.1 (0.4 to 3.4)	4.2 (0.5 to 38.0)
BMI (N)	195	42 (21.5)	175	34 (19.4)	370	76 (20.5)		
< 18.5	15	0 (0)	6	1 (0.6)	21	1 (0.3)	1	
18.5 to 24.99	107	28 (14.3)	96	16 (9.1)	203	44 (11.9)	5.5 (0.7 to 42.4)	
25 to 29.99	60	13 (6.7)	57	10 (5.7)	117	23 (6.2)	4.9 (0.6 to 38.4)	
≥ 30	13	1 (0.5)	16	7 (4.0)	29	8 (2.1)	7.6 (0.8 to 66.5)	
Income per month (N) $	204	45 (22.1)	183	34 (18.6)	387	79 (20.4)		
≥ 250$	49	12 (5.9)	39	5 (2.7)	88	17 (4.4)	1	
< 250$	155	33 (16.2)	144	29 (15.8)	299	62 (16.0)	1.1 (0.6 to 2.0)	

^#^Statistically significant difference P-value < 0.001, ^**+**^Statistically significant difference *P*-value < 0.05

Gender, age, education level, and occupation were multivariable analysis to adjust for confounder

^a^The difference of *O. viverrini* and other IPs infections between SR and BN area was not significantly statistic. GEE: generalized estimating equation, SR: Samran sub-district; BN: Bueng Niam sub-district

### Risk factors associated with *O. viverrini* and IPIs other than *S. stercoralis*

Males were more likely to be infected with *O. viverrini* than females (49/387; 12.7% vs. 30/387; 7.7%). There was a higher prevalence of infection in participants ≥60 years than in other age groups (45/387; 11.6%). Prevalence was highest among participants with primary level or no formal education (68/387; 17.6%). Among the various occupations, participants who worked in agriculture were most likely to be infected (57/387; 14.7%; Table 3). According to the univariate analysis, gender and education level were significantly associated with *O. viverrini* and IPIs other than *S. stercoralis*. Males were 2.4 times more likely to be infected with *O. viverrini* and IPIs than females (cOR 2.4; 95%CI: 1.4 to 3.9, *P*-value < 0.001). Participants with primary level or no formal education were 2.3 times more likely to be infected with *O. viverrini* or IPIs other than *S. stercoralis* than those who had completed grades 7–12 or had a diploma, bachelor’s degree, or higher (cOR 2.3; 95%CI: 0.5 to 10.5, *P*-value = 0.005). Age, occupation, BMI, and income were not significantly associated with *O. viverrini* infection or IPIs other than *S. stercoralis* (*P-*value > 0.05; Table 3). After multivariable analysis to adjust for possible confounders, only gender was a risk factor that was associated with *O. viverrini* infection or IPIs other than *S. stercoralis*. Males were 4.1 times more likely than females to be infected with *O. viverrini* and IPIs other than *S. stercoralis* (aOR 4.1; 95%CI: 2.3 to 7.2, *P-*value < 0.001; Table 3).

## Discussion

This study demonstrated a high prevalence of *S. stercoralis* and *O. viverrini* infections in rural communities in northeast Thailand’s Mueang Khon Kaen district. The overall prevalence of *S. stercoralis* infection as detected by APC of 23.0% was similar to those found in past studies of the same area, reflecting no decline. For instance, a previous survey in northeast Thailand found rates of infection to be 22.7, 23.52, and 28.9% [23–25]. Moreover, the prevalence of *S. stercoralis* infection in this study was higher than in some previous studies. Surveys using the same APC method in northern and southern Thailand found the prevalence to be 15.9% [26] and 20.6% [27], respectively. Other surveys of *S. stercoralis* human infection using FECT, the Kato-Katz technique, and the Harada-Mori technique in northeast Thailand found the prevalence to be from 2.8 to 9.5% [28–30]. The difference in prevalence of *S. stercoralis* among various communities may be due to variations in age, the season of study, the parasitological technique used, personal hygiene practices, and socioeconomic and environmental factors [31–33].

The present study showed various factors (including gender, age, education levels, and occupation) to be significantly associated with *S. stercoralis* infection. We found that males were more likely to be infected with *S. stercoralis* than females (16.7% vs. 6.3%), which was similar to the results found in previous reports [34–39]. This study found that older participants (≥ 60 years) had the highest prevalence of *S. stercoralis* infection (12.2%), which was also similar to the results of previous studies [40]. This is possibly due to older participants having a more prolonged exposure to sources of *S. stercoralis* infection [17, 27]. Most people who were infected with *S. stercoralis* had primary school or no formal education (18.0%). This is consistent with the findings of a previous report [33]. The highest prevalence of *S. stercoralis* infection was in agriculturists (18.0%), which indicates that this group is at high risk of coming in contact with *S. stercoralis* in its infective stage [35, 38]. The reason may be simply because they have greater contact with soil than those in other occupations.

In this study, the prevalence of *O. viverrini* and IPIs other than *S. stercoralis* detected by FECT was 20.4%, which was lower than in a previous study in which it was 37.2% [28] and a national survey in the northeast of Thailand that found 26.0% [30]. However, the prevalence of *O. viverrini* and IPIs other than *S. stercoralis* in this study was higher than in other studies. For instance, a survey using the Kato-Katz technique in northeast Thailand found a prevalence of 7.0% [29], and one in northern Thailand found a prevalence of 5.1% [26]. Moreover, a survey using FECT in northeast Thailand found a prevalence of 5.4% [25]. The differences in prevalences may be due to variations in examination technique, environmental sanitation, socioeconomic factors, and the education level of participants.

The prevalence of *O. viverrini* infection was 19.4%, which was higher than that of other helminths. This finding was similar to those in previous studies. For example, a national survey revealed an *O. viverrini* infection in the northeast in 2001 and 2009 with a prevalence of 15.7 and 16.6%, respectively, which is higher than in other regions [12, 27, 41]. Additionally, a study in rural communities in northeast Thailand showed that *O. viverrini* (26.9%) was a prominent intestinal parasite in this region [28]. Raw fish consumption is common in northeast Thailand and may be related to poverty [27, 28]. A previous report found *Clonorchis sinensis* in central Thailand using a molecular method [42]. *Opisthorchis viverrini* and *C. sinensis* are virtually indistinguishable by egg morphology. This means that microscopic examination may result in an *O. viverrini* infection being misinterpreted for a *C. sinensis* infection. Thus, it is possible that perhaps clonorchiasis and opisthorchiasis may be present in this area. This possibility needs to be tested empirically in the future.

This study found that gender and education levels were significantly associated with *O. viverrini* and IPIs other than *S. stercoralis*. Males had a greater chance of being infected with *O. viverrini* and IPIs other than *S. stercoralis* than females (12.7% vs. 7.7%), which is similar to the findings of previous reports [28]. Most people infected with *O. viverrini* and IPIs other than *S. stercoralis* were primary school graduates or had no formal education (17.6%). These findings were similar to previous reports [28]. Participants ≥60 years old had the highest prevalence of *O. viverrini* and IPI infections other than *S. stercoralis* (11.6%), similar to the results found in previous studies [28, 34]. However, this differs from the results of a national survey, which found the highest infection rates among 40–49-year-olds [27]. The fact that older participants had a high prevalence of *O. viverrini* and *S. stercoralis* may be due to them having more time to be exposed to sources of *S. stercoralis* infection and having consumed a greater amount of raw fish (a source of *O. viverrini* infection).

When considering the prevalence of *S. stercoralis* infection (22.3%) in the SR sub-district (an area in which there was no history of flooding) and the BN sub-district (23.7%; at least one flood per year), the prevalence in the two areas did not differ significantly. This result differs from those of a previous laboratory experiment [43]. Ananmnart et al. [43] demonstrated that prolonged submersion of stool in water was detrimental to the growth and development of *S. stercoralis* rhabditiform larva and suggested that atmospheric conditions and rainfall could possibly affect the growth and development of *S. stercoralis*. This effect is absent in the present study, possibly due to other factors (i.e., poverty, climatic conditions, poor personal hygiene, poor sanitation, population migration, and consumption of raw or semi-raw meat).

## Conclusions

This study demonstrated that, despite the implementation of an intensive national parasite control program [11, 12], a sustainable integrated opisthorchiasis control program [13], as well as a cholangiocarcinoma screening and care program [14] in rural areas of northeast Thailand, the prevalences of *S. stercoralis* and *O. viverrini* have not declined. Another nationwide assessment of intestinal parasitic examination including risk factors, treatment, and prevention should be conducted. Moreover, awareness campaigns and appropriate control programs should be developed to reduce intestinal parasitic infection, especially in agriculturists in rural communities. Additionally, these results should encourage policymakers and public health personnel to improve programs for parasitic control and health promotion.

## Abbreviations

$: US Dollar
95%CI: 95% confidence interval
aOR: adjusted odds ratio
APC: Agar plate culture
BMI: Body mass index
BN: Bueng Niam sub-district
cOR: Crude odds ratio
FECT: Formalin-ethyl acetate concentration technique
GEE: Generalized estimating equation
IPIs: Intestinal parasitic infections
Lao PDR: Lao People’s Democratic Republic
^o^C: Degrees Celsius
SD: Standard deviation
SR: Samran sub-district
WHO: World Health Organization

## References

[1] Jongsuksuntigul P, Intapan PM, Wongsaroj T, Nilpan S, Singthong S, Veerakul S (2003). Prevalence of *Strongyloides stercoralis* infection in northeastern Thailand (agar plate culture detection). J Med Assoc Thail.

[2] Jongwutiwes U, Waywa D, Silpasakorn S, Wanachiwanawin D, Suputtamongkol Y (2014). Prevalence and risk factors of acquiring *Strongyloides stercoralis* infection among patients attending a tertiary hospital in Thailand. Pathog Glob Health.

[3] Nutman TB. Human infection with *Strongyloides stercoralis* and other related *Strongyloides* species. Parasitology. 2016:1–11. 10.1017/S0031182016000834 PMC5563389 27181117

[4] Olsen A, van Lieshout L, Marti H, Polderman T, Polman K, Steinmann P (2009). Strongyloidiasis--the most neglected of the neglected tropical diseases?. Trans R Soc Trop Med Hyg.

[5] Schär F, Trostdorf U, Giardina F, Khieu V, Muth S, Marti H (2013). *Strongyloides stercoralis*: global distribution and risk factors. PLoS Negl Trop Dis.

[6] Beknazarova M, Whiley H, Ross K. Strongyloidiasis: A Disease of Socioeconomic Disadvantage. Int J Environ Res Public Health. 2016;13: pii: E517.10.3390/ijerph13050517 10.3390/ijerph13050517 PMC4881142 27213420

[7] Forrer A, Khieu V, Schar F, Hattendorf J, Marti H, Neumayr A (2017). *Strongyloides stercoralis* is associated with significant morbidity in rural Cambodia, including stunting in children. PLoS Negl Trop Dis.

[8] Grove DI (1996). Human strongyloidiasis. Adv Parasitol.

[9] Sripa B, Kaewkes S, Intapan PM, Maleewong W, Brindley PJ (2010). Food-borne trematodiases in Southeast Asia epidemiology, pathology, clinical manifestation and control. Adv Parasitol.

[10] Bouvard V, Baan R, Straif K, Grosse Y, Secretan B, Ghissassi F (2009). WHO International Agency for Research on Cancer monograph working group. A review of human carcinogens–part B: biological agents Lancet Oncol.

[11] Chongsuvivatwong V, Pas-Ong S, Ngoathammatasna W, McNeil D, Vithsupakorn K, Bridhikitti V (1994). Evaluation of hookworm control program in southern Thailand. Southeast Asian J Trop Med Public Health.

[12] Jongsuksuntigul P, Imsomboon T (2003). Opisthorchiasis control in Thailand. Acta Trop.

[13] Sripa B, Tangkawattana S, Laha T, Kaewkes S, Mallory FF, Smith JF, Wilcox BA (2015). Toward integrated opisthorchiasis control in northeast Thailand: the Lawa project. Acta Trop.

[14] Khuntikeo N, Chamadol N, Yongvanit P, Loilome W, Namwat N, Sithithaworn P, Andrews RH, Petney TN, Promthet S, Thinkhamrop K, Tawarungruang C, Thinkhamrop B (2015). CASCAP investigators. Cohort profile: cholangiocarcinoma screening and care program (CASCAP). BMC Cancer.

[15] Office of Agriculture Economics, Ministry of Agriculture and Cooperatives. 2016. http://www.oae.go.th/assets/portals/1/files/ebook/yearbook59.pdf. Accessed 6 Sep 2017.[In Thai].

[16] Khuntikeo N, Sithithaworn P, Loilom W, Namwat N, Yongvanit P, Thinkhamrop B (2016). Changing patterns of prevalence in *Opisthorchis viverrini* sensu lato infection in children and adolescents in Northeast Thailand. Acta Trop.

[17] Prasongdee TK, Laoraksawong P, Kanarkard W, Kraiklang R, Sathapornworachai K, Naonongwai S (2017). An eleven-year retrospective hospital-based study of epidemiological data regarding human strongyloidiasis in Northeast Thailand. BMC Infect Dis.

[18] Saenna P, Hurst C, Echaubard P, Wilcox BA, Sripa B (2017). Fish sharing as a risk factor for *Opisthorchis viverrini* infection: evidence from two villages in North-Eastern Thailand. Infect Dis Poverty.

[19] Pennsylvania State University, Sample Size Computation for Population Proportion Confidence Interval. 2016. https://onlinecourses.science.psu.edu/stat500/node/31. Accessed 6 Sep 2016.

[20] Koga K, Kasuya S, Khamboonruang C, Sukhavat K, Ieda M, Takatsuka N (1991). A modified agar plate method for detection of *Strongyloides stercoralis*. Am J Trop Med Hyg.

[21] Elkins DB, Haswell-Elkins M, Anderson RM (1986). The epidemiology and control of intestinal helminths in the Pulicat Lake region of southern India. I. Study design and pre- and post-treatment observations on *Ascaris lumbricoides* infection. Trans R Soc Trop Med Hyg.

[22] The Secretariat of the Cabinet. Measures to increase income for low income people. 2016. https://www.mof.go.th/home/Press_release/News2016/154.pdf. Accessed 16 Apr 2018. [In Thai].

[23] Intapan PM, Maleewong W, Wongsaroj T, Singthong S, Morakote N (2005). Comparison of the quantitative formalin ethyl acetate concentration technique and agar plate culture for diagnosis of human strongyloidiasis. J Clin Microbiol.

[24] Sithithaworn J, Sithithaworn P, Janrungsopa T, Suvatanadecha K, Ando K, Haswell-Elkins MR (2005). Comparative assessment of the gelatin particle agglutination test and an enzyme-linked immunosorbent assay for diagnosis of strongyloidiasis. J Clin Microbiol.

[25] Sithithaworn P, Srisawangwong T, Tesana S, Daenseekaew W, Sithithaworn J, Fujimaki Y (2003). Epidemiology of *Strongyloides stercoralis* in north-East Thailand: application of the agar plate culture technique compared with the enzyme-linked immunosorbent assay. Trans Roy Soc Trop Med Hyg.

[26] Nontasut P, Muennoo C, Sa-nguankiat S, Fongsri S, Vichit A (2005). Prevalence of *strongyloide*s in northern Thailand and treatment with ivermectin vs albendazole. Southeast Asian J Trop Med Public Health..

[27] Wongsaroj T, Phatihatakorn W, Ramasoota P, Anamnart W, Kaewpoonsri N, Chiewchanyon B (2008). Epidemiological study of strongyloidiasis in southern Thailand, 2007. J Trop Med Parasitol.

[28] Boonjaraspinyo S, Boonmars T, Kaewsamut B, Ekobol N, Laummaunwai P, Aukkanimart R (2013). A cross-sectional study on intestinal parasitic infections in rural communities, Northeast Thailand. Korean J Parasitol.

[29] Kitvatanachai S, Boonslip S, Watanasatitarpa S (2008). Intestinal parasitic infections in Srimum suburban area of Nakhon Ratchasima Province. Thailand Trop Biomed.

[30] Wongsaroj T, Nithikathkul C, Rojkitikul W, Nakai W, Royal L, Rammasut P. Brief communication (Original). National survey of helminthiasis in Thailand. Asian Biomedicine. 2014:779.

[31] Hotez PJ, Brindley PJ, Bethony JM, King CH, Pearce EJ, Jacobson J (2008). Helminth infections: the great neglected tropical diseases. J Clin Invest.

[32] Hotez PJ, Bundy DAP, Beegle K, Brooker S, Drake L, de Silva N, Jamison DT, Breman JG, Measham AR, Alleyne G, Claeson M, Evans DB (2006). Helminth infections: soil-transmitted helminth infections and schistosomiasis. Disease control priorities in developing countries.

[33] Punsawad C, Phasuk N, Bunratsami S, Thongtup K, Siripakonuaong N, Nongnaul S (2017). Prevalence of intestinal parasitic infection and associated risk factors among village health volunteers in rural communities of southern Thailand. BMC Public Health.

[34] Gonçalves AQ, Junqueira ACV, Abellana R, PCd B, WCM T, Sodré FC (2016). Prevalence of intestinal parasites and risk factors for specific and multiple helminth infections in a remote city of the Brazilian Amazon Rev Soc Bras Med Trop.

[35] Khieu V, Schär F, Forrer A, Hattendorf J, Marti H, Duong S (2014). High prevalence and spatial distribution of *Strongyloides stercoralis* in rural Cambodia. PLoS Negl Trop Dis.

[36] Khieu V, Schär F, Marti H, Bless PJ, Char MC, Muth S (2014). Prevalence and risk factors of *Strongyloides stercoralis* in Takeo Province. Cambodia Parasit Vectors.

[37] Laymanivong S, Hangvanthong B, Insisiengmay B, Vanisaveth V, Laxachack P, Jongthawin J (2016). First molecular identification and report of genetic diversity of *Strongyloides stercoralis*, a current major soil-transmitted helminth in humans from Lao People's Democratic Republic. Parasitol Res.

[38] Steinmann P, Zhou XN, Du ZW, Jiang JY, Wang LB, Wang XZ (2007). Occurrence of *Strongyloides stercoralis* in Yunnan Province, China, and comparison of diagnostic methods. PLoS Negl Trop Dis.

[39] Vonghachack Y, Sayasone S, Bouakhasith D, Taisayavong K, Akkavong K, Odermatt P (2015). Epidemiology of *Strongyloides stercoralis* on Mekong islands in southern Laos. Acta Trop.

[40] Paula F, Costa-Cruz J (2011). Epidemiological aspects of strongyloidiasis in Brazil. Parasitology.

[41] Sithithaworn P, Andrews RH, Nguyen VD, Wongsaroj T, Sinuon M, Odermatt P (2012). The current status of opisthorchiasis and clonorchiasis in the Mekong Basin. Parasitol Int.

[42] Traub RJ, Macaranas J, Mungthin M, Leelayoova S, Cribb T, Murrell KD, Thompson RC (2009). A new PCR-based approach indicates the range of *Clonorchis sinensis* now extends to Central Thailand. PLoS Negl Trop Dis.

[43] Anamnart W, Intapan PM, Pattanawongsa A, Chamavit P, Kaewsawat S, Maleewong W (2015). Effect of dilution of stool soluble component on growth and development of *Strongyloides stercoralis*. Sci Rep.

